# Self-supervised learning for modal transfer of brain imaging

**DOI:** 10.3389/fnins.2022.920981

**Published:** 2022-09-01

**Authors:** Dapeng Cheng, Chao Chen, Mao Yanyan, Panlu You, Xingdan Huang, Jiale Gai, Feng Zhao, Ning Mao

**Affiliations:** ^1^School of Computer Science and Technology, Shandong Business and Technology University, Yantai, China; ^2^Shandong Co-Innovation Center of Future Intelligent Computing, Yantai, China; ^3^College of Oceanography and Space Informatics, China University of Petroleum, Qingdao, China; ^4^School of Statistics, Shandong Business and Technology University, Yantai, China; ^5^Department of Radiology, Yantai Yuhuangding Hospital, Yantai, China

**Keywords:** brain imaging, multiple modal, self-supervised learning, generative adversarial network, auxiliary tasks

## Abstract

Today's brain imaging modality migration techniques are transformed from one modality data in one domain to another. In the specific clinical diagnosis, multiple modal data can be obtained in the same scanning field, and it is more beneficial to synthesize missing modal data by using the diversity characteristics of multiple modal data. Therefore, we introduce a self-supervised learning cycle-consistent generative adversarial network (BSL-GAN) for brain imaging modality transfer. The framework constructs multi-branch input, which enables the framework to learn the diversity characteristics of multimodal data. In addition, their supervision information is mined from large-scale unsupervised data by establishing auxiliary tasks, and the network is trained by constructing supervision information, which not only ensures the similarity between the input and output of modal images, but can also learn valuable representations for downstream tasks.

## Introduction

Brain imaging is crucial in the diagnosis and treatment of neurological diseases. The information provided by a kind of image obtained from an imaging method is limited, and it can only reflect modal information. Generally, it cannot help doctors to make an accurate diagnosis. Modal transfer technology is beneficial to transform different modal images to obtain multimodal information. Combined with multimodal images, it can provide a variety of information regarding diseased tissues or organs, and provides a powerful theoretical basis for accurate diagnosis in clinical medicine. Hence, we present a framework, called BSL-GAN, of self-supervised learning in this paper. This framework not only realizes the transformation among different brain imaging modes, but can also integrate all available information related to the target mode in multi-source modal images to generate any missing modes in a single model. Different from the existing methods of generative adversarial networks (GANs), we introduce an auxiliary network as a new self-supervised constraint that provides information about the target modal data to guide the training of the reconstructed network. In addition, the generated target modal mask vector is used as the target modal data label through self-supervised learning in unsupervised data by an auxiliary network. Finally, we evaluate the performance, generalization performance of the framework self-monitoring learning and cooperative learning on experiments with 1.5T images and 3T image datasets, and demonstrate the valuable performance of the framework for downstream tasks in experiments with missing modal data compared with other methods. The results show that our proposed framework has advantages.

## Related work

There are a lot of medical imaging modality data in the field of medical imaging. In addition, Zhao et al. (2020, 2022) used functional connectivity networks to explore the discriminative information provided by different brain networks. Cheng et al. (2021) used generative adversarial networks to realize the conversion from EEG modality data to fMRI modality data. According to whether the data need to be paired or manually labeled, brain imaging modality migration methods can be divided into two types: supervised learning-based methods and unsupervised learning-based methods.

The methods based on supervised learning require that the input data must be marked or paired, but they take a lot of manpower and cost. Edmund and Nyholm (2017) report many methods for the generation of substitute CT images for MRI-only radiotherapy. Han (2017) used the method of minimizing the voxel difference between CT and MR images, which strictly aligns the acquired MR images and CT images, because this method uses the deep convolution neural network with paired data. Zhao et al. (2017) used the improved (Ronneberger et al., 2015) to synthesize MR from CT images, and then used the synthesized MR images for brain segmentation based on CT. However, minimizing the loss of voxel direction between the synthesized image and the reference image during training may result in blurred output. Nie et al. (2017) proposed a method of combining voxel loss with countermeasure loss in generating a countermeasure network to obtain clearer results. A parallel work by Bi et al. (2017) also proposed a GAN framework to synthesize positron emission tomography (PET) images. Isola et al. (2017) proposed a pix2pix framework to conduct image-to-image translation. Ben-Cohen et al. (2017) combined a fully convolutional network (Long et al., 2015) and pix2pix model to output the target results, and mixed the two outputs to generate PET images from CT images. Although the combination of voxel direction loss and countermeasure direction loss solves the problem of fuzzy output, the voxel direction loss still depends on a lot of paired images.

Most medical institutions have quite a lot of unpaired data, which are scanned for different purposes and different radiotherapy techniques. Zhu et al. (2017) proposed a framework named cycle-GAN to solve the problem of image-to-image translation. This framework not only combined voxel loss and antagonism loss, but also put forward the concept of cyclic consistency loss, so that training can be carried out without relying on paired data. Wolterink et al. (2017) synthesized CT images from MR images by using unpaired data. The above loop-based method alleviates the dependence of paired data to a certain extent. However, there could be excessive deformation in the generated images, and this may affect their clinical applications. Hence, aligned data or auxiliary tasks are still necessary for these tasks. Although these methods have shortcomings, their advantages are worth learning. Therefore, in this paper, the setting of objective function also includes voxel loss and cyclic consistency loss, benefiting from stable optimization of supervised learning and large-scale datasets of unsupervised learning.

## BSL-GAN

Although the existing GAN-based methods have greatly improved the quality of synthesized images, these images are often found to be distorted or blurred. The main reason is that these methods implement a loss function calculated from the pixel-level difference between the generated image and the real reference image. As far as we know, no work directly uses feature-level constraints to guide the decoder to obtain a better learning generator.

Knowledge distillation (Kim and Rush, 2016; Liu et al., 2019), extracts general, moderate, and sufficient knowledge from the “teacher” network to guide the “student” network, and the experienced “teacher” network can guide the generation of decoders in the network at the functional level. We need a network with a strong representation ability to guide the decoder better.

Therefore, the classification model (Simonyan and Zisserman, 2015) can be pre-trained on the large-scale natural image dataset (Deng et al., 2009), and enough feature maps with a strong representation ability can be extracted to realize knowledge transfer. However, for medical images that are more complex than natural images, it is difficult to directly use the knowledge derived from natural images to guide the generator network. In fact, it is also impossible to obtain large-scale medical image datasets for pre-training. To sum up, medical image synthesis should be better than natural image synthesis. Therefore, we propose a self-supervised learning framework for medical image processing, namely, BSL-GAN.

The BSL-GAN framework can be supervised by the input image itself, and has a similar architecture to the generator in the GAN-based method. Since the (Rumelhart et al., 1986; Vincent et al., 2008; Kingma and Welling, 2014) only works on a single domain and is faster than a generator that learns the mapping function between two different domains, it is also easy to converge. Its powerful self-representation ability finds wide use in other tasks (such as feature dimensionality reduction). Therefore, we borrow the auto-encoder network to guide the decoder network at the feature level, which is better than learning from the reconstructed image only through backpropagation with pixel-level loss.

**Figure 1:**
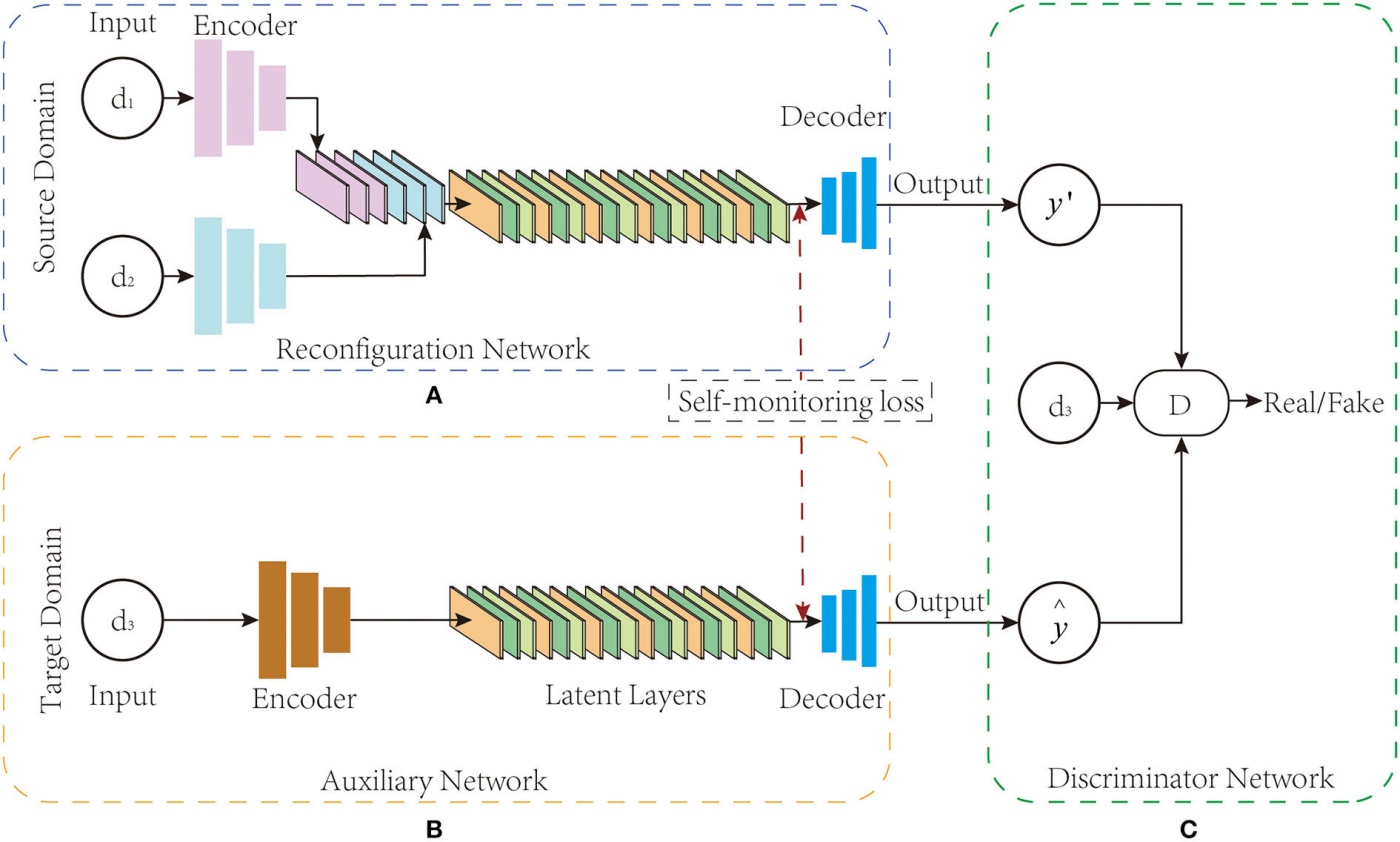
**(A–C)** BSL-GAN framework structure. BSL-GAN realizes the conversion between 1.5T MR images and 3T MR images.

As shown in Figure 1, the BSL-GAN framework proposed in this paper consists of three key parts: reconstruction network R, auxiliary network P, and discriminator network D. All these three components train data in an end-to-end manner. First, the function of reconstruction network R is to realize the transformation from source domain modal data to target domain modal data. It designs a generator structure like GAN, and correspondingly, it includes three components: encoder, converter, and decoder. Among them, in the encoder of R, it consists of several branches, where each branch corresponds to a kind of modal data; in the converter of R, it is composed of a batch normalization (BN) layer and a latent layer. In the decoder of R, it has only one branch and finally outputs the converted modal data. Second, the auxiliary network P refers to the network structure of self-encoder. The encoder and decoder have only one branch, and only the target image is input, while the target modal data vector is output. The discriminator network D has three branch inputs: the vector of the target modal image generated by the auxiliary network, the reconstructed image generated by the reconstruction network, and the real target modal image.

In the training stage, the reconstruction network R encodes the input image into the common potential feature space. The converter fuses the deep features of the connections from the input images to extract their complementary information for generating images through the decoder. Auxiliary network P adopts the form of self-encoder, which is only trained by the target image. Once trained, the feature map extracted from the decoder of the auxiliary network P is used to guide the optimization of the decoder of the reconstruction network R. In the testing stage, the auxiliary network and the discriminator network are removed, and only the reconstruction network is used to translate images from multiple source domains to target domains. For different input combinations from different source domains, the BSL-GAN framework can generate images of missing modes through a single unified model.

### Reconfiguration network

Inspired by the existing image translation methods, this paper develops an encoder-decoder network architecture like the GAN generator structure for reconstruction network R (Noh et al., 2015). As shown in Figure 1A, the reconstruction network R consists of three parts: multi-branch encoder *E*_*R*_, converter *T*_*R*_, and decoder *De*_*R*_. The number of branches in the encoder network is determined by the total number of input modes, and each branch consists of three convolution layers. Particularly, the converters in the converter network are composed of a batch standardization layer (Ioffe and Szegedy, 2015) and a latent layer, and the latent layer is composed of six residual blocks (He et al., 2016), each of which is in the form of Conv-BN-ReLu-BN. For each residual block, their input is the output of the last batch normalization layer.

During training, the reconstruction network R inputs the source domain modal data into the encoder, and then encodes the source domain image into the common potential feature space through the potential layer in the converter network. Finally, the target domain image is reconstructed through the deconvolution layer of the decoder. In the test, the auxiliary network and discriminator network are removed, and only the reconstruction network R is used to reconstruct the image source domain to the target domain. For images from the source domain, the BSL-GAN framework can generate images with missing modes.

Suppose that there are two kinds of datasets: source domain *O* and target domain *A*. There are two kinds of modal data {*d*_1_, *d*_2_} in the source domain *O*, and only one kind of modal data {*d*_3_} in the target domain. Given the input image *x*_*d*_*O, i*__, (*i*∈1, 2) from the source domain *O* and the input image *y*_*d*_*A*, 3__ from the target domain *A*, the encoder branch *E*_*R, i*_(*i*∈1, 2) in the reconstruction network R encodes the input image to the converter branch, and the converter encodes the source domain image into the common potential feature space as $f_{i}^{T_{R}}$:


(1) $$\begin{array}{r} f_{i}^{T_{R}}=E_{R,\;i}\left(x_{d_{O,\;i}}\right),i\in 1,2 \end{array}$$

where *E*_*R, i*_(·) denotes the forward calculation process of convolution network, and *i*_*i*_ denotes modality. The converter *T*_*R*_ extracts the fused complementary information $f_{i}^{T_{R}}$ from the concatenated coding features. The decoder *De*_*R*_ extracts the feature map $f_{i}^{T_{R},\;De_{R}}$ from $f_{i}^{T_{R}}$ as follows:


(2) $$\begin{array}{r} f_{i}^{T_{R},\;De_{R}}\text{=}De_{R,\;i}\left(f_{i}^{T_{R}}\right) \end{array}$$

where *i* denotes the*i*−*th* layer of decoder network. *De*_*R, i*_(·) denotes the forward computation process of the decoder in the reconstruction network R.

### Auxiliary network

This paper introduces an auxiliary network into the proposed new framework which serves as the supervision constraint of the BSL-GAN framework and provides information about target modal data to guide the training of the reconstruction network to improve the traditional brain imaging modal migration method based on unsupervised learning. In addition, self-supervised learning is performed in unsupervised data through the auxiliary network, and the generated target modal mask vector is used as the target modal data label. As described in the reconstruction network, the self-encoder is trained to reconstruct the input itself, which ensures the strong representation ability of the self-encoder in the same domain. Considering this, one of the key objectives of the proposed framework is to guide the decoders in the reconstruction network through the decoders in the auxiliary network. Here, the same network architecture as the reconstructed network is used, except that multiple branches are merged into a single branch. Therefore, the self-encoder framework is utilized in the auxiliary network P.

As shown in Figure 1B, the auxiliary network P can be regarded as a self-encoder network, which mainly consists of an encoder *E*_*P*_, a latent layer *l*_*P*_, and a decoder *De*_*P*_. In addition, the modal mask vector obtained from the auxiliary network training is used as the target modal label, which guides the reconstruction network of the BSL-GAN framework to transform images from various input modal images into any lost modal images during the training process. For a given input ground-truth image *y*_*d*_*A*, 3__ from the target domain, the encoder *E*_*P*_ of the auxiliary network P encodes it into the latent space *l*^*P*^:


(3) $$\begin{array}{r} l^{P}\text{=}E_{P}\left(y_{d_{A,\;3}}\right) \end{array}$$

Similar to the reconstruction network R, the potential features of *l*^*P*^ are used to feed the decoder *De*_*P*_ of the auxiliary network P and extract the feature map $f_{i}^{P,\;De_{P}}$:


(4) $$\begin{array}{r} f_{i}^{P,\;De_{P}}=De_{P,\;i}\left(l^{P}\right) \end{array}$$

where *De*_*P, i*_(·) denotes the forward calculation process of the decoder in network P, and *i* denotes the *i*−*th* layer of the decoder network. The image reconstructed by the reconstruction network P, the target modal label *y*′ = *P*(*y*) generated by the auxiliary network, and the ground-truth image *y*_*d*_*A*, 3__ are inputs into the discriminator network D which together train its discrimination ability.

### Discriminator network

As shown in Figure 1C, BSL-GAN uses “PatchGAN” (Isola et al., 2017) in the discriminator network. Unlike distinguishing whether each pixel of the input image is real or fake, this discriminator network tries to classify each patch in the input image that determines whether it is true or false. Such a patch-level discriminator punishes the structural loss on the patch scale and has fewer parameters than the whole image discriminator.

In the training of the discriminator, the ground-truth image *y* of the target domain, the image reconstructed by the reconstruction network, and the modal mask vector obtained by the auxiliary network training are taken as inputs. The modal mask vector here is also the target modal label, which has the same size matrix as the training image. For each target modal label, the elements of each matrix in the modal mask vector share the same value.

### Network losses

The structure of the BSL-GAN framework has been introduced above, and then the loss function involved in the framework is mainly introduced. In the BSL-GAN framework, this paper designs three kinds of losses: self-monitoring loss, discriminator loss, and multi-branch generator loss.

#### Self-monitoring loss

In the proposed BSL-GAN framework, an auxiliary network is constructed as a self-monitoring constraint to guide the reconstruction network training. Therefore, in the proposed BSL-GAN framework, the self-monitoring loss between the auxiliary network and the reconstructed network is designed. Different from the traditional method of brain imaging modal migration based on GAN, BSL-GAN is supervised not only by pixel-level loss, but also by feature-level loss. As described above, the auxiliary network p is trained by the target image itself. When training with the reconstruction network R, the auxiliary network P will better simulate the distribution of target images than the reconstruction network R. Therefore, we introduce the feature mapping of decoder *De*_*P*_ to guide decoder *De*_*R*_. Given the three kinds of modal data of two datasets, our proposed framework can generate another missing modal data from the other two modal data. Assuming that {*d*_3_} is generated from {*d*_1_, *d*_2_}, the loss *L*_*SLC*_ from self-supervision can be defined as follows:


(5) $$\begin{array}{r} L_{d_{3}}^{SLC}\;=\;\overset{n}{\underset{i}{\sum }}{\left||f_{i,\;d_{3}}^{P,\;De_{P}}-f_{i,\;d_{1},\;d_{2}}^{P,\;De_{R}}|\right|}_{2} \end{array}$$

where || · ||_2_ denotes *l*_2_−*norm*, *De*^*i*^(·) denotes the output of the *i*−*th* layer in the decoder network, and *n* represents the number of convolution layers in the decoder networks *De*_*P*_ and *De*_*R*_.

#### Discriminator loss

The discriminator is used to predict whether the input image is true or false. As mentioned above, the auxiliary network P can estimate the distribution of the target domain more accurately than the reconstructed network R. We not only merged the fake image ŷ reconstructed by R, but also merged the pseudo image *y*′ generated by the auxiliary network P for training the decoder network and the real image *y*. Therefore, the discriminator impairment *L*_*D*_ can be calculated as follows:


(6) $$\begin{array}{r} L_{D}\left(y,ŷ,y^{\prime }\right)\;=\;E_{y:P_{y}}\left[\log\left(D\left(y\right)\right)\right]\\ \;\text{+}\lambda_{1}E_{ŷ:P_{ŷ}}\left[\log\left(1-D\left(ŷ\right)\right)\right]\\ \;\text{+}\left(1-\lambda_{1}\right)E_{y^{\prime }:P_{y^{\prime }}}\left[\log\left(1-D\left(y^{\prime }\right)\right)\right] \end{array}$$

where λ_1_∈(0, 1) denotes the value of the auxiliary network and the weight network.

#### Multi-branch generator loss

Since our model can generate any missing mode from the other three modes, the generator loss is the sum of four different input combinations. We take *l*_1_ loss as pixel-level loss to supervise the reconstruction network R and auxiliary network P to avoid the blurring effect caused by *l*_2_ loss. When *m*_1_ is the target mode, the generator loss $L_{m_{1}}^{G,\;R}$of the reconstruction network R and the generator loss of the auxiliary network $L_{m_{1}}^{G,P}$can be given as follows:


(7) $$\begin{array}{r} L_{m_{1}}^{G,\;R}=E_{x:P_{x}}\left[{\left||R\left(x_{m_{1}|m_{2},\;m_{3}}\right)-y|\right|}_{1}\right]\\ L_{m_{1}}^{G,\;P}=E_{x:P_{x}}\left[{\left||P\left(x_{m_{1}}\right)-y|\right|}_{1}\right] \end{array}$$

Therefore, the multi-branch generator loss *L*^*G, R*^ of reconstruction network R and the generator loss *L*^*G, P*^ of the auxiliary network P can be written as:


(8) $$\begin{array}{r} L_{G}=L_{m_{k}}^{G,\;R}+L_{m_{k}}^{G,\;P},\;k\in \left\{1,\;2,\;3\right\} \end{array}$$

where *k* means that *m*_*k*_ is the target mode. Our total loss is formulated as follows:


(9) $$\begin{array}{r} L\;=\;L_{SLC}\;+\;L_{D}\;+\;\lambda_{2}\cdot L_{G} \end{array}$$

where λ_2_ = 10. *L*_*SLC*_ means self-supervision, *L*_*D*_ means discriminator loss, and *L*_*G*_ means the total generator loss.

## Experiments

We verify the effectiveness of the BSL-GAN framework through experiments in four different scenarios:

- Supervised learning performance and cooperative learning performance test: control test without auxiliary task and control test with an auxiliary task, and single-branch input and multi-branch input.
- Generalization performance test: conversion test from 1.5T MR images to 3T MR images.
- Performance test of synthetic missing modes: There are three modes of MR images obtained by a 1.5T scanner, namely, T1-FLAIR, T2-FLAIR, and T2-TRF, and any missing modes are generated from the other two modes.

In addition, we compare the BSL-GAN framework with several latest brain imaging mode conversion methods. In this section, we will describe the dataset, experimental implementation details, model performance evaluation, and qualitative and quantitative results to prove the effectiveness of the brain imaging modality migration method based on supervised learning.

### Datasets

Our dataset is obtained from Yuhuangding Hospital of Yantai City, which was scanned by 1.5T MRI and 3T MRI scanners. This dataset consists of 22 subjects. The study was approved by the institutional review board of Yantai Yuhuangding Hospital and the Ethics Committee of Shandong Technology and Business University while patient informed consent was waived. Every subject has three magnetic resonance imaging modes: T1-FLAIR, T2-FLAIR, and T2-TRF. The size of each MRI image is 256 × 256 × 1, and the voxel size is 1 × 1 × 1.

In all of the experiments, 80% of the subjects were randomly selected as the training set. The remaining 20% of subjects were used as the test set. We verify the performance of the model by changing the network input and output modalities:

- Supervised learning performance test: in the 1.5T scanning field, the input and output of tasks without assistance are the same, that is, the T1-FLAIR image and T2-FLAIR image are inputs, and the T2-TRF image is the output.
- Cooperative learning performance test: in the 1.5T scanning field, the T1-FLAIR image or T2-FLAIR image is input in a single branch, the T1-FLAIR image and T2-FLAIR image are inputs in multiple branches, and the T2-TRF image is output if single branch input and multi-branch input have the same output.
- Generalization performance test: T2-FLAIR image in 1.5T scanning field is input, and T2-FLAIR image in 3T scanning field is output.
- Synthetic missing modal performance test: three modal images, namely, T1-FLAIR, T2-FLAIR, and T2-TRF, are obtained by a 1.5T scanner, and the missing third modal image is generated by inputting two modal images.

### Experimental details

**Figure 2:**
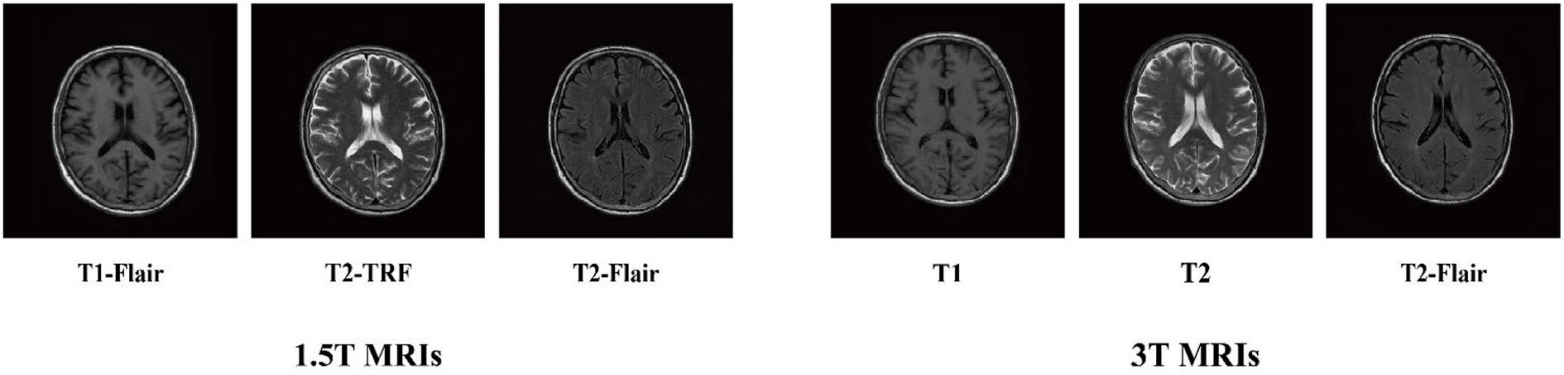
1.5T MRIs and 3T MRIs.

We used MicroDicom visualization software to visualize the two types of imaging data and obtained 1.5T and 3T axial slice images of T1-FLAIR, T2-FLAIR, and T2-TRF, respectively (Figure 2). Each subject involved 20 axial slices. Their pixel sizes were the same as 256 × 256 × 1. Next, we used AMD Ryzen 7 4800H and NVIDIA GeForce RTX 2060 processor for 2e5 iterations. The whole training process takes about 40 h. According to the slice-based scanning principle of medical images, we cut the 3D medical images into multiple slices and utilize them to train the proposed method. All the images used in our experiments are spatially aligned. Then we convert each 2d slice to grayscale. In our experiments, the parameter λ_1_ is set to 0.5 and λ_2_ is set to 10. We used structural similarity index (SSIM) and feature similarity index (FSIM) as evaluation criteria to objectively evaluate the quantitative score of translated images. All real images from the target modality were used as reference datasets. SSIM and FSIM scores of translated images were used for quantitative evaluation.

### Characteristic similarity index

Peak signal-to-noise ratio (PSNR), mean square error (MSE), structural similarity index measure (Wang et al., 2004 SSIM), and feature similarity index (Zhang et al., 2011. FSIM) are selected as evaluation criteria. The PSNR, MSE, and SSIM have been introduced before the publishing of this article, and the FSIM index is mainly introduced in this study. This index holds that all pixels in a picture do not have the same importance. For example, pixels at the edge of an object are more important to define the structure of an object than pixels in other background areas.

Based on some studies in psychology and brain science, it is found that Fourier waves with different frequencies have the same phase, which often corresponds to visually recognizable important features. This means that feature information can be extracted from some consistent phases. However, phase consistency (Zhang et al., 2011. PC) is relatively invariant to image changes, which helps to extract stable features in images, but sometimes image changes do affect perception, so it needs to be compensated by gradient magnitude (GM). PC and GM are used in FSIM to complement each other. FSIM is obtained by coupling PC and GM terms:


(10) $$\begin{array}{r} FSIM=\frac{\sum_{x\in \omega }S_{L}\left(x\right)PC_{m}\left(x\right)}{\sum_{x\in \omega }PC_{m}\left(x\right)} \end{array}$$

where *PC*_*m*_(*x*) = max(*PC*_1_(*x*), *PC*_2_(*x*)), *PC*(*x*)∈(0, 1], andω means the whole image pixel domain. *PC*_1_(*x*) and *PC*_2_(*x*) mean the PC values of the first and the second image, respectively. Intuitively, for a given location *x*, if either of the two images has a significant PC value, it implies that this position *x* will have a high impact on human visual system when evaluating the similarity between the two images. Therefore, we define *S*_*L*_(*x*) as follows:


(11) $$\begin{array}{r} S_{L}\left(x\right)={\left[S_{PC}\left(x\right)\right]}^{\alpha }\cdot \;{\left[S_{G}\left(x\right)\right]}^{\beta } \end{array}$$

where α = β = 1. The similarity measure *S*_*PC*_(*x*) and *S*_*G*_(*x*) can be calculated as follows:


(12) $$\begin{array}{r} S_{PC}\left(x\right)=\frac{2PC_{1}\left(x\right)\cdot PC_{2}\left(x\right)+T_{1}}{PC_{1}^{2}\left(x\right)+PC_{2}^{2}\left(x\right)+T_{1}} \end{array}$$

(13) $$\begin{array}{r} S_{G}\left(x\right)=\frac{2G_{1}\left(x\right)\cdot G_{2}\left(x\right)+T_{2}}{G_{1}^{2}\left(x\right)+G_{2}^{2}\left(x\right)+T_{2}} \end{array}$$

where *G*_1_(*x*) and *G*_2_(*x*) represent the GM values of the first and the second image, respectively. *T*_1_ and *T*_2_ are positive constants that depend on the dynamic range of PC and GM values. For the calculation of *PC*(*x*) and *G*(*x*), please refer to Zhang et al. (2011).

### Comparison method

We compared BSL-GAN with the following methods:

- Pix2pix: Pairing data is trained using a combination of L1 distance and antagonism loss.
- StarGAN (Choi et al., 2018): The above pix2pix framework is applicable to the modal migration of paired data, that is, the transformation from one domain to another. However, the pix2pix framework is not applicable when modal data in more than two fields need to be migrated. StarGan can transform multi-domain modal data into desired target modal data.

### Analysis of experimental results

The BSL-GAN framework proposed in this paper has been verified by experiments. In this paper, the BSL-GAN framework is compared with the traditional pix2pix framework based on supervised learning and the StarGAN framework with multi-branch input, which proves that the performance of our proposed framework is excellent. Next, we will evaluate and analyze the performance of self-supervised learning and cooperative learning, generalization and synthesis of missing modal data, and prove the feasibility and effectiveness of the BSL-GAN framework.

#### Performance analysis of self-supervised learning and cooperative learning

This paper divides the experiment into two parts to verify the effectiveness of self-supervised learning and multi-branch cooperative learning based on the BSL-GAN framework. First, we set up a control task group for the self-supervised learning performance of the framework. One is the framework for removing the auxiliary network from the framework, and the other is the framework with the help of the auxiliary network. The precondition is that the inputs of these two sets of frames are the same. The T1-FLAIR image and T2-FLAIR image acquired under the 1.5T scanner will be used as the inputs, and the T2-TRF image will be used as the target output.

**Figure 3:**
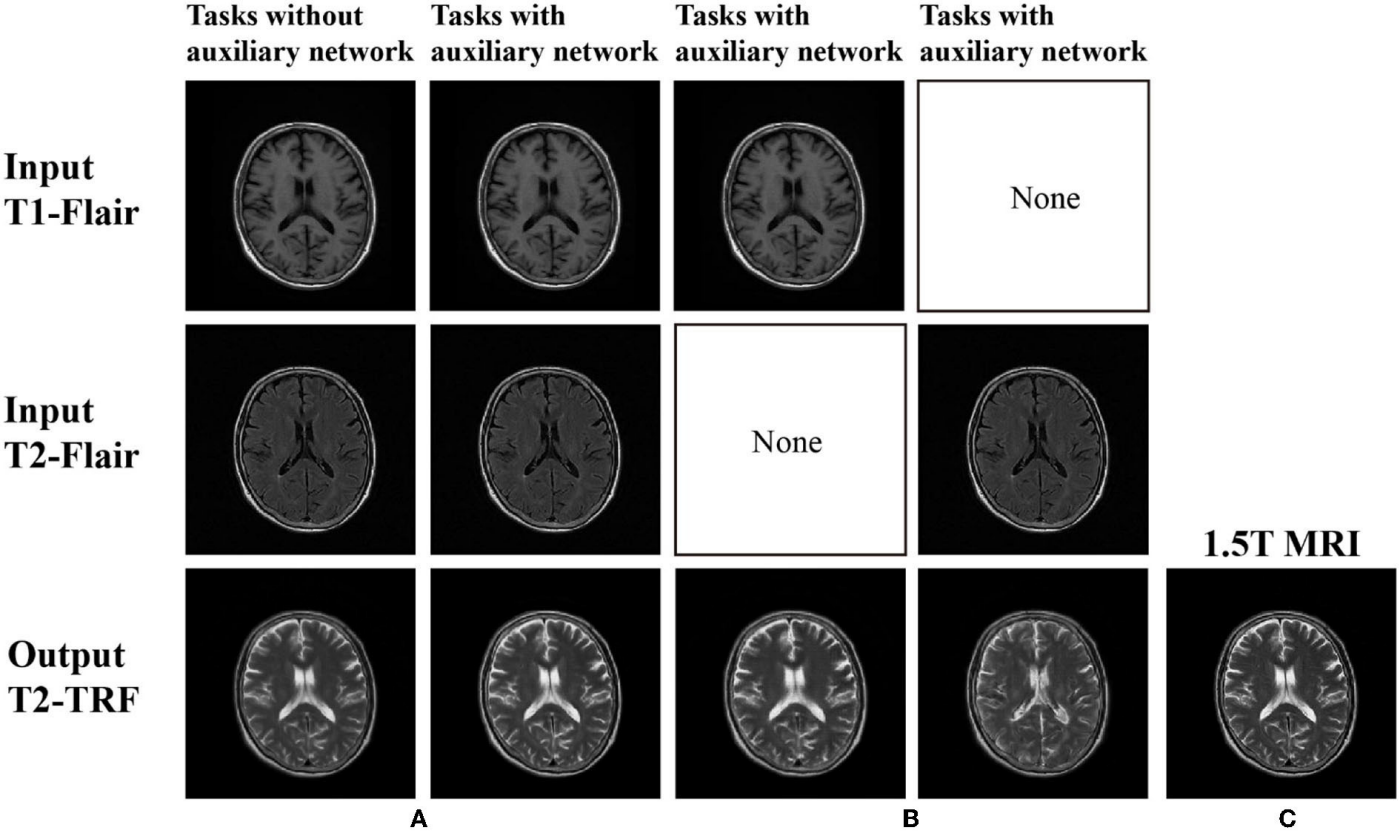
**(A)** There is no self-monitoring constraint in image generation; **(B)** Images generated under self-supervision constraints; and **(C)** 1.5T MRI (ground truth).

In Figure 3, the images generated without auxiliary tasks are fuzzy in appearance and lack useful anatomical details. However, this framework obtains a clearer output image and generates more anatomical details like the reference target image with the help of auxiliary tasks. According to Table 1, the scores obtained by MSE, PSNR, SSIM, and FSIM also indicate that the framework under the guidance of auxiliary tasks has achieved the highest results.

**Table 1:** Self-supervised learning performance index and cooperative learning performance index.

	**Tasks without auxiliary network**	**Tasks wit auxiliary network**	**T1-FLAIR**	**T2-FLAIR**
MSE	171.69 ± 20	80.58 ± 20	117.16 ± 20	107.68 ± 20
PSNR	25.78 ± 2	29.07 ± 1	27.44 ± 2	27.81 ± 2
SSIM	0.80 ± 0.03	0.92 ± 0.01	0.87 ± 0.03	0.91 ± 0.02
FSIM	0.87 ± 0.03	0.93 ± 0.02	0.89 ± 0.03	0.91 ± 0.02

In addition, for the analysis of the multi-branch cooperative learning performance of the BSL-GAN framework, this paper also sets up a control task group. The task group is implemented in the framework of an auxiliary network. One group only takes the T1-FLAIR image acquired under the 1.5T scanner as input, and the other group only takes the T2-FLAIR image acquired under the 1.5T scanner as input. T2-TRF image acquired by 1.5T scanner is output as the target. These two groups of tasks are compared with the above-mentioned tasks with an auxiliary network with T1-FLAIR and T2-FLAIR as inputs.

**Figure 4:**
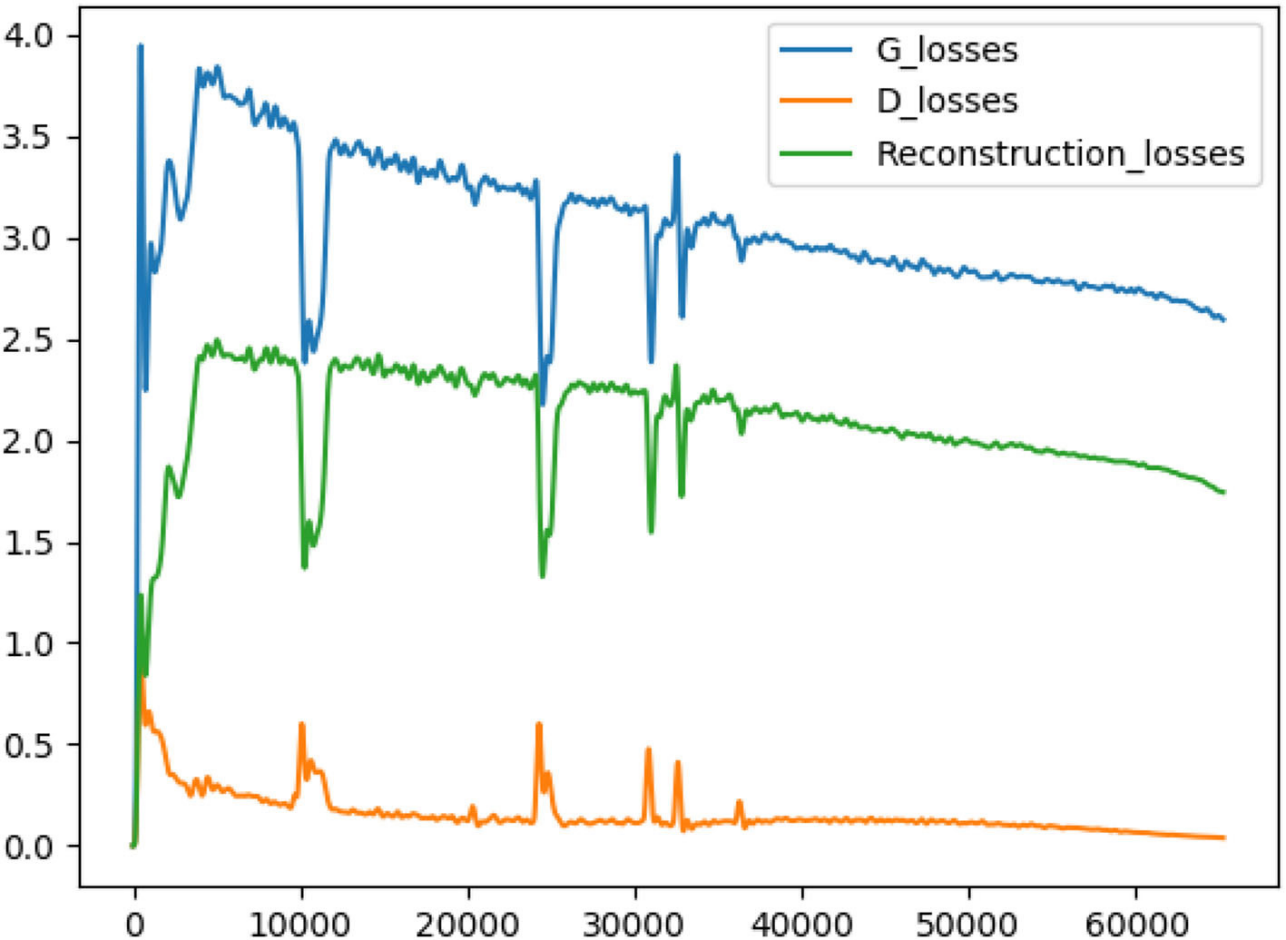
The blue line represents the generator loss, the yellow line represents the discriminator loss, and the green line represents the reconstructed loss.

As shown in Figure 3, if there is no multi-branch input to realize cooperative learning, the generated output image has fuzzy anatomical details. These are the output images and target reference images generated by naked eye observation. Moreover, the loss of each iteration is shown in Figure 4, and the loss of the discriminator converges step by step. Finally, this paper also uses SSIM and FSIM to evaluate the results quantitatively. As shown in Table 1, the proposed BSL-GAN framework under the guidance of auxiliary tasks reaches 0.9250 in SSIM and 0.9264 in FSIM, which are much higher than 0.8380 and 0.8853 in the group without auxiliary tasks and higher than the score of only one branch input.

#### Generalization performance analysis

The above experiments verify the superiority of self-supervised learning performance and cooperative learning performance of the framework proposed in this paper, and the following experiments verify the generalization performance of the framework proposed in this paper. Through the research in this paper, it is found that the existing modal data migration framework based on GAN in the field of medical imaging is a single branch input, that is, the modal data in one field is converted into the modal data in another field. Therefore, this paper reduces the input branch of the reconstruction network to one and simplifies the BSL-GAN framework to prove that it can be implemented well in this case. Similarly, this experiment can further prove the self-supervised learning performance of BSL-GAN.

**Figure 5:**
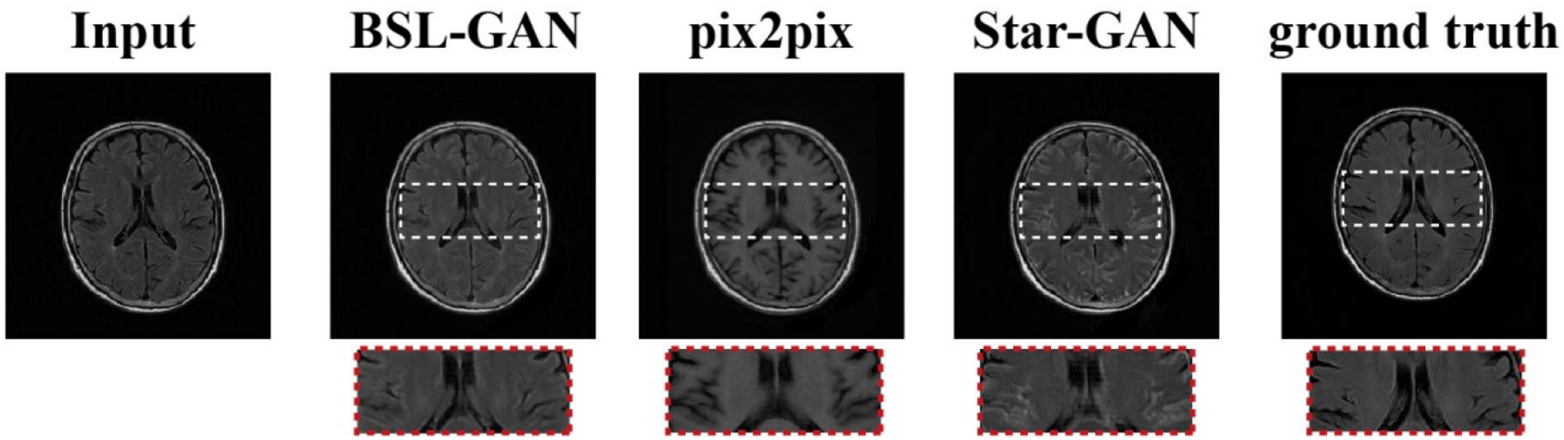
Task of 1.5T MRI to 3T MRI.

As shown in Figure 5, in clinical practice, the strong magnetic field possessed by a 3T magnetic resonance scanner may affect the health of patients with metal implants, while a 1.5T magnetic resonance scanner is considered safe and non-invasive. Therefore, in the experiment, the T2-FLAIR modal image obtained from a 1.5T scanning domain is transformed into the T2-FLAIR modal image obtained from a 3T scanning domain. Qualitative evaluation showed that Pix2Pix and StarGAN had a poor perceived appearance and large deformation around the skull. The simplified framework of BSL-GAN can obtain qualified pseudo 3T MR modal images with more accurate and clearer skull contour. For quantitative evaluation, this paper compares the synthesized 3T MR modal image with the real 3T MR modal image and calculates SSIM and FSIM scores. As shown in Table 2, the BSL-GAN framework obtains the highest SSIM and FSIM scores, which is superior to other methods. The experimental results further verify the effectiveness of the proposed feature-level self-supervised learning method.

**Table 2:** Comparison of self-monitoring constraint performance under different models: Comparison of scores between single-branch input and multi-branch input in task method with auxiliary network.

	**BSL-GAN**	**pix2pix**	**StarGAN**
SSIM	0.92 ± 0.01	0.89 ± 0.02	0.86 ± 0.02
FSIM	0.95 ± 0.02	0.93 ± 0.02	0.90 ± 0.02

#### Performance analysis of synthetic missing modal data

In this paper, the BSL-GAN framework is compared with two popular GAN-based methods (pix2pix and StarGAN). We assume that one of the modal datasets in the1.5T scanning field is missing, and the remaining two modal data in the 1.5T scanning field are used as input for training. Then, the results of synthetic missing modes are analyzed. Pix2Pix and StarGAN need a single input. T2-FLAIR image is used as input in this paper, because it provides more information about tumor lesions than the other three methods.

**Figure 6:**
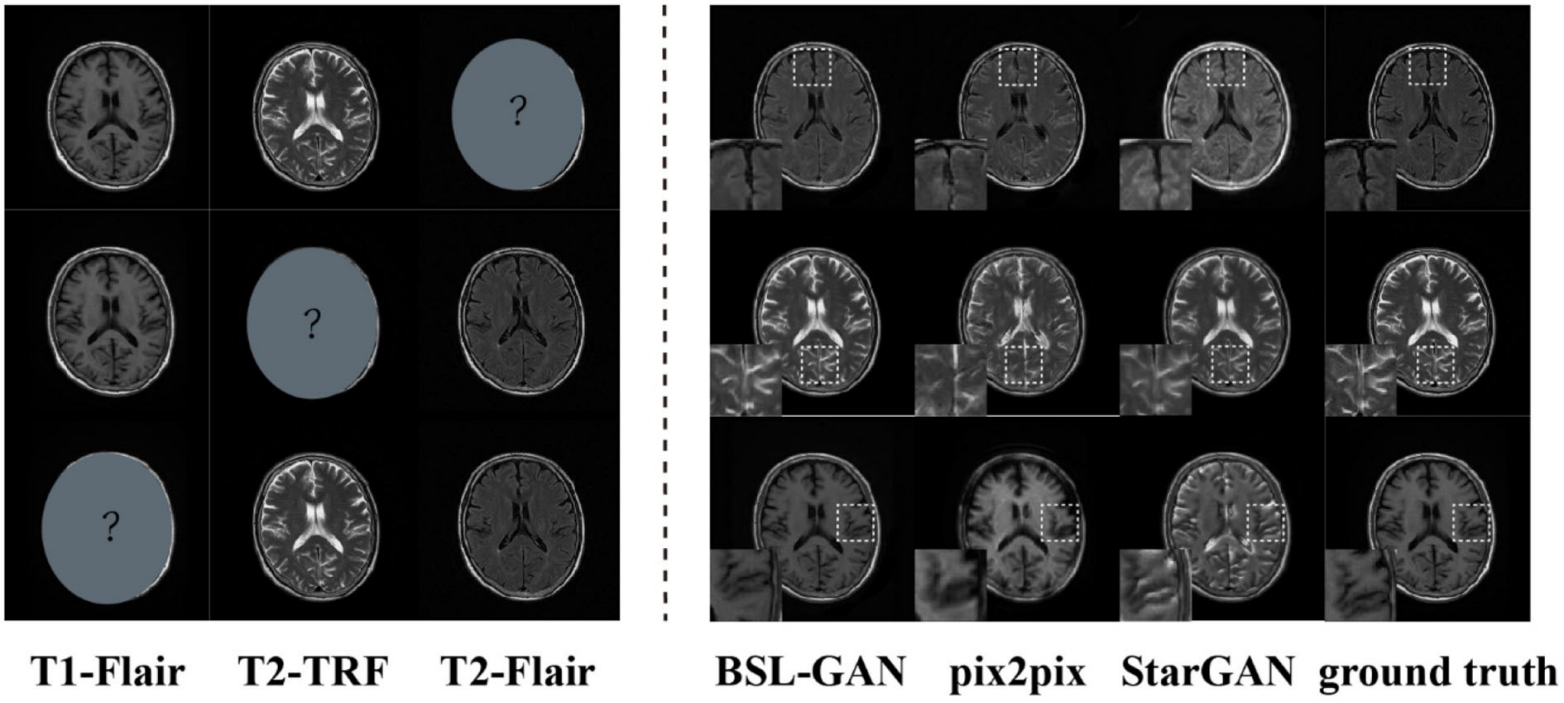
Images synthesized *via* missing modal data and the experimental results of other methods.

As shown in Figure 6, the output reconstructed image of the proposed BSL-GAN framework is very similar to the reference image, and the soft tissue details and boundary texture are clear, which is superior to other methods. In Figure 6, Pix2pix and StarGAN reconstruct 3T MR images from 1.5TMR images, but the output results show a poor perceived appearance, and the details are unclear.

For quantitative evaluation, SSIM and FSIM scores are shown in Table 3, and these scores are calculated from reconstructed images and reference images. Because other methods based on GAN only use pixel-level loss, they cannot learn the accurate distribution of target modes at the feature level, which reduces the quantitative SSIM and FSIM scores. By comparison, the BSL-GAN proposed in this paper can estimate any missing modes from other available modes in a unified single model and has excellent qualitative and quantitative performance, which can be more efficient in the testing stage.

**Table 3:** SSIM and FSIM scores of the proposed method are compared with Pix2Pix and StarGAN.

	**T1-FLAIR**		**T2-TRF**		**T2-FLAIR**	
	**SSIM**	**FSIM**	**SSIM**	**FSIM**	**SSIM**	**FSIM**
BSL-GAN	0.94 ± 0.01	0.95 ± 0.01	0.90 ± 0.01	0.92 ± 0.01	0.91 ± 0.01	0.93 ± 0.01
pix2pix	0.90 ± 0.02	0.92 ± 0.02	0.89 ± 0.02	0.91 ± 0.02	0.88 ± 0.02	0.91 ± 0.02
StarGAN	0.83 ± 0.03	0.88 ± 0.03	0.87 ± 0.03	0.91 ± 0.03	0.86 ± 0.03	0.89 ± 0.03

## Conclusion

Magnetic resonance imaging is widely used as an important means to study brain diseases. The magnetic resonance intensity has developed from 0.5T to 1.5T or 3T, which is widely used now. Compared with 1.5T magnetic resonance imaging, 3T magnetic resonance imaging provides better contrast and higher resolution images, which provide potential value for the diagnosis and treatment. However, susceptibility artifacts often occur when 3T magnetic resonance scanners have strong magnetic fields, and some patients with implants and foreign bodies cannot use them, which leads to the loss of related brain imaging modality data for these patients.

In this paper, we introduce a self-monitoring method that uses an auxiliary network to realize self-supervised learning which is based on unsupervised learning to guide the decoders in the reconstruction network and synthesize reconstructed images with higher quality. In addition, the modal mask vector obtained by the auxiliary network reconstruction can be used as the target modal label, so that our self-monitoring framework can generate any missing modes and further ensure its generalization. Although the proposed BSL-GAN achieves better performance than other advanced technologies, it has several limitations. For example, in the training stage, the proposed framework needs more computing resources and computing time. In the future, we will explore more efficient network architecture to deal with more realistic and complex applications.

## Data availability statement

The raw data supporting the conclusions of this article will be made available by the authors, without undue reservation.

## Ethics statement

The studies involving human participants were reviewed and approved by the Institutional Review Board of Yantai Yuhuangding Hospital and the Ethics Committee of Shandong Technology and Business University. Written informed consent for participation was not required for this study in accordance with the national legislation and the institutional requirements.

## Author contributions

DC: conceptualization, methodology, and writing—review and editing. CC: conceptualization, software, writing—original draft, methodology, formal analysis, investigation, and validation. MY: writing—review, editing, and validation. PY, XH, JG, FZ, and NM: writing—review and editing. All authors contributed to the article and approved the submitted version.

## Funding

This research was supported by the National Natural Science Foundation of China (No: 62176140).

## Conflict of interest

The authors declare that the research was conducted in the absence of any commercial or financial relationships that could be construed as a potential conflict of interest.

## Publisher's note

All claims expressed in this article are solely those of the authors and do not necessarily represent those of their affiliated organizations, or those of the publisher, the editors and the reviewers. Any product that may be evaluated in this article, or claim that may be made by its manufacturer, is not guaranteed or endorsed by the publisher.
